# Liquid chromatography–tandem mass spectrometry assay for the simultaneous determination of apalutamide and its active metabolite N-desmethyl apalutamide, and its application in real-world patients with castration-resistant prostate cancer

**DOI:** 10.3389/fphar.2025.1510583

**Published:** 2025-05-19

**Authors:** Yuxuan Zhu, Lu Chen, Wenqiang Cheng, Dingyuan Yang, Lijuan Zhang, Jun Li

**Affiliations:** ^1^ Department of Pharmacy, Personalized Drug Therapy Key Laboratory of Sichuan Province, Sichuan Academy of Medical Science & Sichuan Provincial People’s Hospital, University of Electronic Science and Technology of China, Chengdu, China; ^2^ Guanghan People’s Hospital, Guanghan, China; ^3^ Department of Urology, The Second People’s Hospital of Yibin, Yibin, China; ^4^ Department of Urology, Chengdu Second People’s Hospital, Chengdu, China; ^5^ Department of Urology, Sichuan Provincial People’s Hospital, University of Electronic Science and Technology of China, Chengdu, China

**Keywords:** apalutamide, N-desmethyl apalutamide, therapeutic drug monitoring, LC–MS/MS, castration-resistant prostate cancer, human plasma

## Abstract

**Background:**

Apalutamide is used in the treatment of castration-resistant prostate cancer. A simple, specific, selective, and effective liquid chromatography–tandem mass spectrometry method for quantifying apalutamide and its active metabolite concentration in human plasma was developed and validated according to the FDA and EMA validation guidelines.

**Methods:**

A total of 24 patients diagnosed with desmoplasia-resistant prostate cancer (NM-CRPC) were recruited. Blood samples were drawn after 4 weeks’ administration of apalutamide at a dose of 180 mg once daily to ensure steady-state blood levels were achieved. Apalutamide and N-desmethyl apalutamide were analysed by quantitative liquid chromatography tandem mass spectrometry to measure the concentrations among individuals and the effect on the baseline level of prostate-specific antigen (PSA) and adverse events.

**Results:**

The linear range, precision, accuracy, matrix effect, recovery, carryover, and stability were appropriate according to the FDA and EMA validation guidelines. The apalutamide blood concentration range of the 24 patients was 0.517–7.27 μg/mL, and the median value was 4.92 μg/mL. The N-desmethyl apalutamide blood concentration range was 1.78–8.32 μg/mL, and the median value was 5.71 μg/mL. The median serum PSA level decreased from 61.03 (range 0.57–885.93) ng/mL at baseline to 0.970 (range 0.01–47.9) ng/mL at week 4.

**Conclusion:**

Therapeutic drug monitoring can help evaluate the individual differences between patients taking apalutamide. A dose of 180 mg could reduce the baseline PSA level significantly (p < 0.05), and the incidence of skin rash was less compared to that of a dose of 240 mg.

## 1 Introduction

Prostate cancer ranks among the three most prevalent cancers in men, accounting for 14% (903,500) and 6% (258,400) of all male cancer-related deaths worldwide (Jemal et al., 2011). Although initial-stage prostate cancer can be successfully treated with surgery, radiotherapy, and androgen-deprivation therapy (ADT), nearly all patients with advanced prostate cancer eventually progress to castration-resistant prostate cancer (CRPC) (Rasool et al., 2019), developing resistance, either intrinsic or acquired, to first-line ADT and other hormonal therapies (Yu et al., 2019). The initial treatment for metastatic prostate cancer currently involves androgen elimination *via* orchiectomy or the administration of luteinising hormone-releasing hormone (LHRH) agonists/antagonists, often combined with anti-androgenic modalities. Clinically, prostate cancer is classified as advanced or localised, and its treatment varies from surveillance to androgen-deprivation or local-radical therapy (Parkin et al., 2001). Approximately 70%–80% of patients with advanced prostate cancer experience symptom reduction through androgen deprivation, yet most tumours recur to androgen intolerance within 2 years, rendering them incurable (Andersson et al., 1997). After one–three years of treatment, almost all tumours that initially responded to deprivation therapy advanced to CRPC (Nishan et al., 2020). A range of therapeutic agents are available for CRPC, including androgen receptor (AR) inhibitors, 5α-reductase inhibitors, immunosuppressive therapy, aldehyde-keto reductase inhibitors, and steroid sulphate esterase inhibitors. Among them, AR inhibitors, such as bicalutamide, flutamide, oestrogens, or ketoconazole, have been the main choice for prostate cancer treatment in recent years. However, they do not independently correlate with an improvement in overall survival (Lodde et al., 2010).

Apalutamide, a novel AR antagonist, belongs to the second generation of highly selective AR antagonists, exhibiting over five-fold greater affinity for AR than first-generation bicalutamide (Clegg et al., 2012). On 14 February 2018, the FDA approved the marketing of apalutamide, marking the FDA’s first new antitumour drug approval based on the clinical endpoint of metastasis-free survival (MFS) (Rathkopf and Scher, 2018). Different disease progressions and therapeutic needs call for distinct therapeutic goals. An effective pharmacological target for the treatment of prostate cancer using androgen antagonists is the inhibition of androgen binding to androgen receptors (Hotte and Saad, 2010). The advent of AR inhibitor apalutamide has extended the survival period for patients with advanced prostate cancer (Nuhn et al., 2019; Suzuki et al., 2008).

AR antagonist therapy comes along with a wide range of mild-to-severe adverse events (AEs), often of the augmented type, which may be exacerbated by drug overexposure. Intolerable AE and toxicity frequently lead to dose reductions or treatment discontinuations, endangering treatment efficacy. In contrast to intravenous chemotherapy, non-adherence might be an additional factor leading to varying drug exposure. One strategy to prevent under- or over-exposure of drug concentrations and monitor adherence is therapeutic drug monitoring (TDM). For apalutamide, the exposure–safety analysis demonstrated that within the observed exposure range in apalutamide-treated patients, the exposure–treatment emergent adverse events relationship was only statistically significant for skin rash and weight loss. Patients treated with 240 mg once daily who experience them may benefit from a dose reduction (Perez-Ruixo et al., 2020). Therefore, exploring the relationships between exposure–response, exposure–safety, and dose reductions in patients and, consequently, PK targets have the potential benefit for individualising the appropriate dose and therapeutic drug monitoring.

To further investigate concentration-dependent effects and the potential use of TDM for apalutamide in daily clinical treatment, in this study, we present the development and validation of a simple, specific, selective, and effective LC–MS/MS method for the quantitative determination of apalutamide in human plasma. This method was successfully applied for therapeutic drug monitoring in 24 patients with enervation-resistant prostate cancer treated with long-term apalutamide.

## 2 Materials and methods

### 2.1 Materials and chemicals

In this study, we utilised a 1260 High Performance Liquid Chromatograph (HPLC; Agilent, USA), a SCIEX 5500QTRAP Mass Spectrometer (SCIEX, USA), an IKA VORTEX genius3 vortex mixer (IKA, Germany), a Hettich UNIVERSAL 320 R High Speed Centrifuge (Hettich, Germany), and a Mettler Toledo MS205DU analytical balance (Mettler Toledo, Switzerland).

Apalutamide, batch no. A16HS191729, was purchased from Med Mol, China; N-desmethyl apalutamide, batch no. 154154, was purchased from Target Mol, U.S.A.; apalutamide-d^4^, batch no. 85392, was purchased from MedChemExpress, U.S.A.; acetonitrile, HPLC grade, was purchased from Fisher, U.S.A.; methanol, HPLC grade, was purchased from Fisher, U.S.A.; DMSO (purity, 99.5%) was purchased from BioFroxx, Germany; and formic acid, HPLC grade, was purchased from Chengdu Cologne Chemical Co. Ultrapure water was obtained using a Milli-Q system (Waters Millipore, MA, United States).

### 2.2 LC–MS/MS conditions

The chromatographic separation of apalutamide, N-desmethyl apalutamide, and the internal standard (IS) in processed samples was achieved using an Ultimate XB-C18 column (50 × 4.6 mm, 5 μm; Welch Corporation, Shanghai, China) maintained at 40°C ± 1°C. The binary mobile phase system consisted of reservoirs of 55% solvent A (0.1% formic acid in acetonitrile) and 45% solvent B (0.1% formic acid in water) running at a flow rate of 0.4 mL/min. The injection volume was 1 μL, and the injection plate temperature was set at 4°C.

**FIGURE 1:**
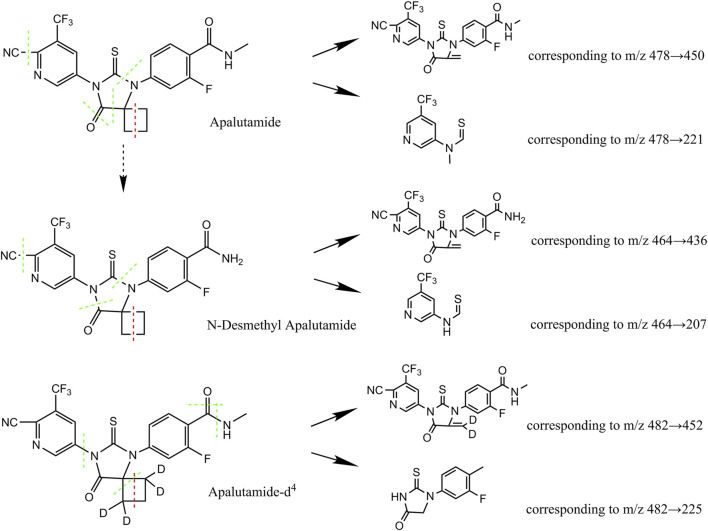
Chemical structures of apalutamide and metabolite N-desmethyl apalutamide quantified by the LC–MS/MS method developed.

Quantitation was achieved through MS/MS detection in positive electrospray ionisation (ESI+) mode for apalutamide, N-desmethyl apalutamide, and the IS using a Sciex API 5500 mass spectrometer (Foster City, CA, USA) equipped with a Turbo ion-spray interface at 500°C and 5,500 V ion spray voltage. The common parameters were set as follows: 40 psi curtain gas (CUR), 50 psi nebuliser gas (GAS1), 50 psi auxiliary gas (GAS2), and collision gas (CAD): medium. The compound-specific parameters including declustering potential (DP), entrance potential (EP), collision exit potential (CXP), and collision energy (CE) for apalutamide, N-desmethyl apalutamide, and the IS were set at 260 V, 10 V, 15 V, and 33 eV; 260 V, 10 V, 15 V, and 34 eV; and 260 V, 10 V, 15 V, and 33 eV, respectively. The dwell time was set to 50 m. Ion detection was performed in the multiple-reaction monitoring (MRM) mode. Quantitation of apalutamide, N-desmethyl apalutamide, and the IS was achieved by monitoring the precursor Q1 → product ions Q3 ions at m/z 478 → 450 (quantifier), 478 → 221 (qualifier); 464.1 → 435.9 (quantifier), 464.1 → 207.1 (qualifier); and 482 → 451.9 (quantifier), 482 → 225.2 (qualifier), respectively (Figure 1). The analytical data were processed using Analyst software (version 1.6.2).

### 2.3 Preparation of calibration curve (CC) standards and quality control (QC) samples

Primary stock solutions of apalutamide, N-desmethyl apalutamide, and apalutamide-d^4^ for the preparation of the calibration curve (CC) and QC samples were prepared separately by weighing. The individual primary stock solutions of apalutamide, N-desmethyl apalutamide, and apalutamide-d^4^ were 1 mg/mL, 1 mg/mL, and 165 μg/mL, respectively, which were prepared in DMSO:methanol (0.2:99.8, v/v). These stock solutions were stored at −80°C. Prior to analysis, dilution was carried out to prepare working standards of 500 μg/mL from the apalutamide and N-desmethyl apalutamide stock solutions using DMSO:methanol (0.2:99.8, v/v). The IS apalutamide-d^4^ control stock solution was pipetted and diluted with DMSO:methanol (0.2:99.8, v/v) to obtain 5 μg/mL of the IS working solution.

Blank human plasma was spiked with prior calibration standard working solution to obtain the highest calibration standard. The highest QC sample was established by spiking the prior separately prepared working solution to blank human plasma. The remaining target calibrators and QC samples were obtained by serial dilution with blank human plasma. Finally, the calibrator concentrations of plasma were 20, 10, 5, 2.5, 1, 0.5, 0.25, 0.1, 0.05, and 0.025 μg/mL for apalutamide and N-desmethyl apalutamide, respectively. The QC samples prepared for apalutamide and N-desmethyl apalutamide were as follows: 25 ng/mL (lower limit of quantification QC (LLOQ QC)), 50 ng/mL (low QC (LQC)), 3.5 μg/mL (medium QC (MQC)), and 17.5 μg/mL (high QC (HQC)). All QC samples were stored at −80°C.

### 2.4 Sample preparation

All plasma samples were stored in a refrigerator at −80°C until analysis. Calibration standards, QC samples, and patient samples were thawed at room temperature and vortexed for 3 min at room temperature. An amount of 50 μL of plasma sample was combined with 10 μL of IS working solution. The mixture was vortexed for 30 s. Subsequently, 100 μL of acetonitrile (0.1% formic acid) was added to precipitate the proteins. The sample was vortexed for an additional 5 min and then centrifuged at low temperature at 4°C (14,000 r/min) for 5 min. The clear supernatant (100 μL) was transferred into vials, and 1.0 μL was injected into the LC–MS/MS system for analysis.

### 2.5 Quantitative methods

Based on the chromatographic peak area values of apalutamide, N-desmethyl apalutamide, and the IS apalutamide-d^4^, the ratios of the analyte’s peak area to that of the IS were calculated to quantify the concentration of the analyte in the samples and the QC samples within the concentration range of 0.025 μg/mL to 20 μg/mL of the CCs.

### 2.6 Method validation

The method validation was performed based on different international guidelines including the Food and Drug Administration (FDA) and European Medicines Agency (EMA) recommendations (Smith, 2012; US-FDA, 2022). Apalutamide and N-desmethyl apalutamide were assessed for specificity, linearity, precision, accuracy, extraction recovery (ER), matrix effects (ME), carryover, and stability in human plasma.

### 2.7 Clinical application

Blood samples were collected from patients who provided written informed consent to participate in the clinical study of apalutamide. The research protocol was approved by the local Medical Ethics Committee. This investigation included adult male patients diagnosed with non-metastatic desmoplasia-resistant prostate cancer (NM-CRPC), who were treated with apalutamide in conjunction with androgen deprivation at our facility. Apalutamide was administered at a dose of 180 mg once daily. Apalutamide was administered to patients orally for at least 4 weeks to ensure that steady therapeutic blood levels were achieved. Blood samples were collected half an hour before drug administration. The samples were centrifuged at 4,000 r/min for 5 min, and the supernatant was stored in a −80°C refrigerator until analysis.

### 2.8 Statistics

Statistical analyses were performed using SPSS (version 25.0, Inc., Chicago, IL, USA). Averages, standard deviations (SD), coefficient of variation (CV), and medians (interquartile range (IQR)) were used to present continuous data. After measurement, if the variables obeyed normal distribution, the data were expressed as x ± s, and t-test was used; if the variables did not obey normal distribution, the data were expressed as M (P25, P75), and the Wilcoxon Mann–Whitney U-test was used. Two-tailed *p*-values <0.05 were regarded as statistically significant.

## 3 Results and discussion

### 3.1 Development of the chromatography and mass spectrometry method

Previous reports on the HPLC method with UV detector for separating plasma samples of apalutamide in mice indicated low analytical efficiency, where the LLOQ was 209 ng/mL (Suresh et al., 2018; Zakkula et al., 2019). In this study, the retention times of apalutamide on the column were 5.45 min and 4.49 min, resulting in a chromatographic run time of 7.0 min, using the LC–MS/MS method. We compared the effects of different mobile phase ratios on the response and retention times. Various mobile phase ratios were tested, and the ratio of solution B (0.1% formic acid in water) in the mobile phase was increased from 20% to 50%, which led to increased retention times of apalutamide and N-desmethyl apalutamide. The highest analyte response and suitable retention time were observed with a 45% aqueous phase. Additionally, different proportions of formic acid (0.1%–0.2%) were tested, showing little difference in retention time but a notable increase in the peak area of 0.1% formic acid in water. Therefore, isocratic elution of an acetonitrile (0.1% formic acid)/water (0.1% formic acid) ratio of 55/45, providing suitable retention time, was selected as the mobile phase, considering the sensitivity and analysis time.

N-desmethyl apalutamide, the primary metabolite of apalutamide, results from demethylation of the amino side chain. Because the cyano side chain, amino side chain, and imidazole ring are susceptible to cleavage, information on the associated characteristic fragment ions can be obtained, as shown in Figure 1. Apalutamide produced characteristic fragment ions at m/z 450 and m/z 221, whereas N-desmethyl apalutamide produced characteristic fragment ions at m/z 436 and m/z 207.

### 3.2 Specificity and selectivity

**FIGURE 2:**
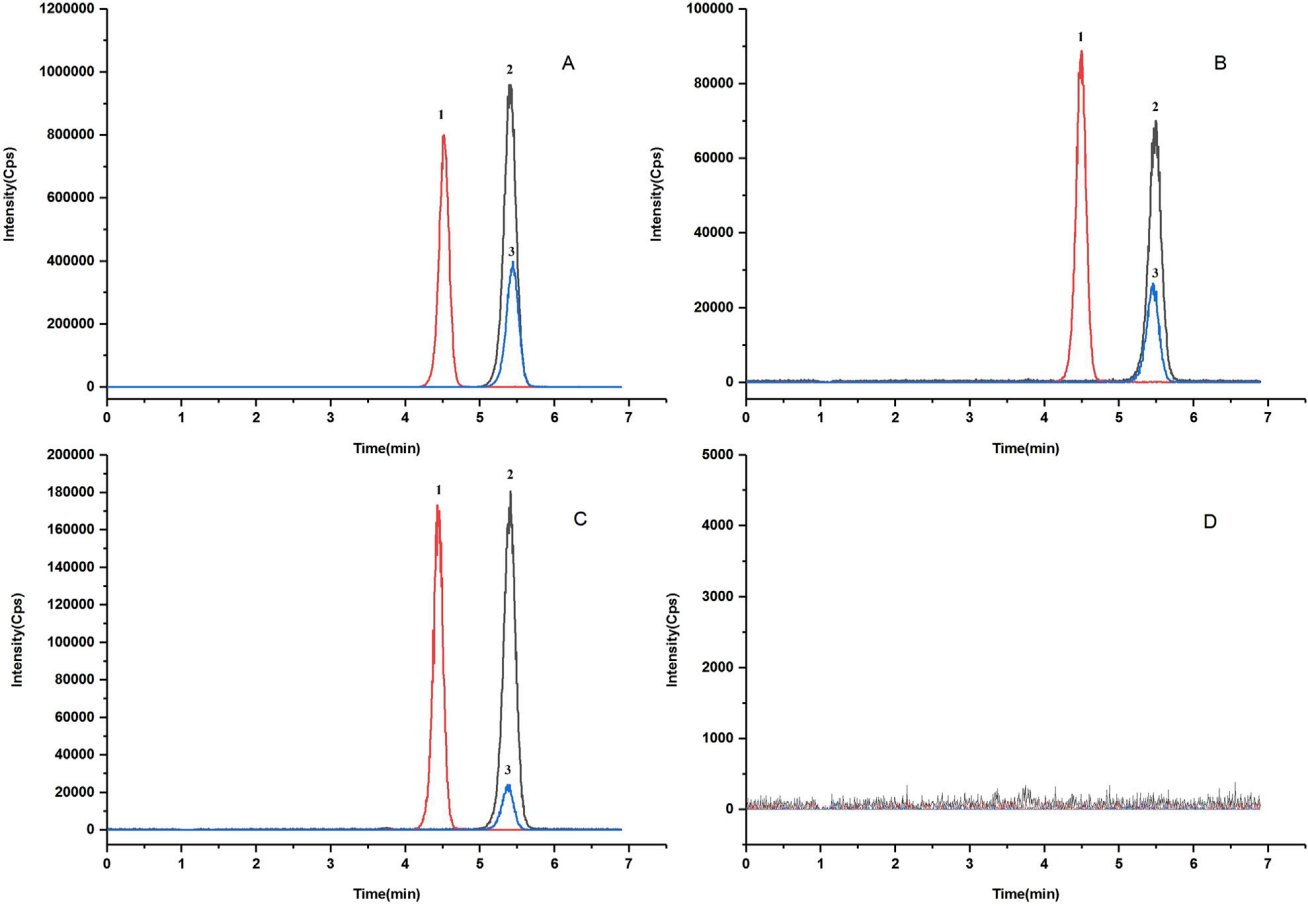
Representative MRM chromatograms of N-desmethyl apalutamide (1), apalutamide (2), and IS (3) from **(A)** standard solutions; **(B)** a plasma spiked with apalutamide (5.0 μg/mL), N-desmethyl apalutamide (5.0 μg/mL), and IS (5.0 μg/mL); **(C)** a real human plasma sample collected half an hour before drug administration of 180 mg apalutamide; **(D)** a blank sample without any analyte and IS added.

The retention times for apalutamide and N-desmethyl apalutamide were 5.45 min and 4.49 min, respectively; the chromatographic peaks displayed good peak shapes, and endogenous substances in plasma did not interfere with the determination of apalutamide and N-desmethyl apalutamide, as demonstrated in Figure 2.

### 3.3 Linearity

Linear regression equations for the concentrations of apalutamide and N-desmethyl apalutamide were obtained using their respective concentrations of apalutamide and N-desmethyl apalutamide in plasma samples as the horizontal coordinates, the ratios of the peak areas of apalutamide and N-desmethyl apalutamide to the IS as the vertical coordinates, and a weighted regression using a weighting factor of W = 1/x^2^. The CC exhibited good linearity within the concentration range of 0.025–20 μg/mL (apalutamide: y = 2.21x + 0.0029, *r* ^2^ = 0.9991; N-desmethyl apalutamide: y = 1.38x + 0.000824, *r* ^2^ = 0.9989). The LLOQ for both apalutamide and N-desmethyl apalutamide was 0.025 μg/mL.

### 3.4 Intra- and inter-day precision and accuracy

We determined the intra- and inter-day precision and accuracy for the four concentration levels (0.025, 0.050, 3.5, and 17.5 μg/mL). Six samples of each concentration were measured on three consecutive days, and intra- and inter-day precision and accuracy were calculated based on the CCs of the same day. All calculated concentrations were within the accepted variable limits.

The precision test showed that the method’s repeatability and reproducibility were reliable. The coefficient of variation (CV) for intra-day and inter-day precision of apalutamide were in the range of 1.7%–4.7% and 3.2%–9.8%, respectively, and those for N-desmethyl apalutamide were in the range of 2.0%–6.7% and 3.2%–10.9%, respectively. The relative errors for intra- and inter-day accuracy of apalutamide were in the range of −5.8%–1.7% and −4.0% to −6.5%, respectively, and those for N-desmethyl apalutamide were in the range of −2.2%–10.1% and −0.8%–1.7%, respectively. The results are summarised in Table 1.

**TABLE 1:** Precision and accuracy for the determination of apalutamide and N-desmethyl apalutamide in human plasma.

QC level	Intra-day (n = 6)			Inter-day (n = 6 × 3)		
	Mean (μg/mL)	Accuracy RE (%)	Precision CV (%)	Mean (μg/mL)	Accuracy RE (%)	Precision CV (%)
Apalutamide						
LLOQ-0.025 μg/mL	0.0254	1.7	1.7	0.0239	−4.4	9.8
LQC-0.05 μg/mL	0.048	−3.3	4.7	0.048	−4.0	6.4
MQC-3.5 μg/mL	3.30	−5.8	2.6	3.29	−6.1	3.7
HQC-17.5 μg/mL	16.85	−3.7	3.5	16.37	−6.5	3.2
N-desmethyl apalutamide						
LLOQ-0.025 μg/mL	0.0275	10.1	6.7	0.0253	1.4	10.9
LQC-0.05 μg/mL	0.053	5.7	4.4	0.051	1.7	7.6
MQC-3.5 μg/mL	3.42	−2.2	2.0	3.47	−0.8	3.8
HQC-17.5 μg/mL	17.82	1.8	2.3	17.65	0.9	3.2

### 3.5 Matrix effect, extraction recovery, and carryover

Table 2 shows the matrix effect of apalutamide, N-desmethyl apalutamide, and the IS. The matrix effect was evaluated using the IS normalised MF, with CV < 5.24% for apalutamide and CV < 6.45% for N-desmethyl apalutamide. The extraction recoveries of apalutamide, N-desmethyl apalutamide, and IS were ranged from 104.4% to 109.7%, 103.5% to 110.4%, and 104.5%, respectively. There was no carryover from residues in samples.

**TABLE 2:** Matrix effect and recovery of apalutamide, N-desmethyl apalutamide, and IS in human plasma (n = 6).

QC level	Analyte MF mean (%)	IS MF mean (%)	IS-normalised MF (n = 6)			Extraction recovery (n = 6)
			Mean	SD	CV (%)	Mean ± SD (%)
Apalutamide						
LQC-0.05 μg/mL	92.3	97.4	0.95	0.05	5.24	109.7 ± 8.23
MQC-3.5 μg/mL						108.8 ± 7.51
HQC-17.5 μg/mL	98.4	100.3	0.98	0.03	3.54	104.4 ± 2.98
N-desmethyl apalutamide						
LQC-0.05 μg/mL	90.1	97.4	0.92	0.05	5.05	110.4 ± 9.78
MQC-3.5 μg/mL						110.4 ± 4.70
HQC-17.5 μg/mL	99.0	100.3	0.99	0.06	6.45	103.5 ± 2.64
Apalutamide-d^4^						
5.0 μg/mL						104.5 ± 4.97

### 3.6 Stability

Sample stability was examined according to the requirements involved in the actual sample determination process. The results showed that apalutamide and N-desmethyl apalutamide were stable at room temperature (25°C) for at least 24 h and in an auto-sampler at 4°C for at least 24 h in extract, and they were stable through three freeze–thaw cycles and during storage at −80°C for 30 days in plasma. Table 3 presents the results of the study.

**TABLE 3:** Stability of apalutamide and N-desmethyl apalutamide in human plasma (n = 6).

Stability tests		Room temperature for 24 h		Auto-sampler for 24 h		Three freeze–thaw cycles		−80°C 30 days	
		Accuracy RE (%)	Precision CV (%)	Accuracy RE (%)	Precision CV (%)	Accuracy RE (%)	Precision CV (%)	Accuracy RE (%)	Precision CV (%)
Apalutamide stability (n = 6)	LQC-0.05 μg/mL	0.7	3.5	−2.1	4.6	−5.4	5.1	4.8	6.8
	MQC-3.5 μg/mL	2.8	1.7	6.3	7.0	7.5	3.6	11.6	3.8
	HQC-17.5 μg/mL	3.5	2.7	8.7	3.1	10.0	4.6	12.9	1.7
N-desmethyl apalutamide stability (n = 6)	LQC-0.05 μg/mL	0.4	6.3	−6.6	6.7	0.6	4.4	10.5	11.3
	MQC-3.5 μg/mL	−0.4	2.4	0.1	5.0	4.7	3.6	12.7	4.6
	HQC-17.5 μg/mL	0.4	5.4	1.5	5.9	7.2	5.5	14.9	3.3

### 3.7 Clinical application

**FIGURE 3:**
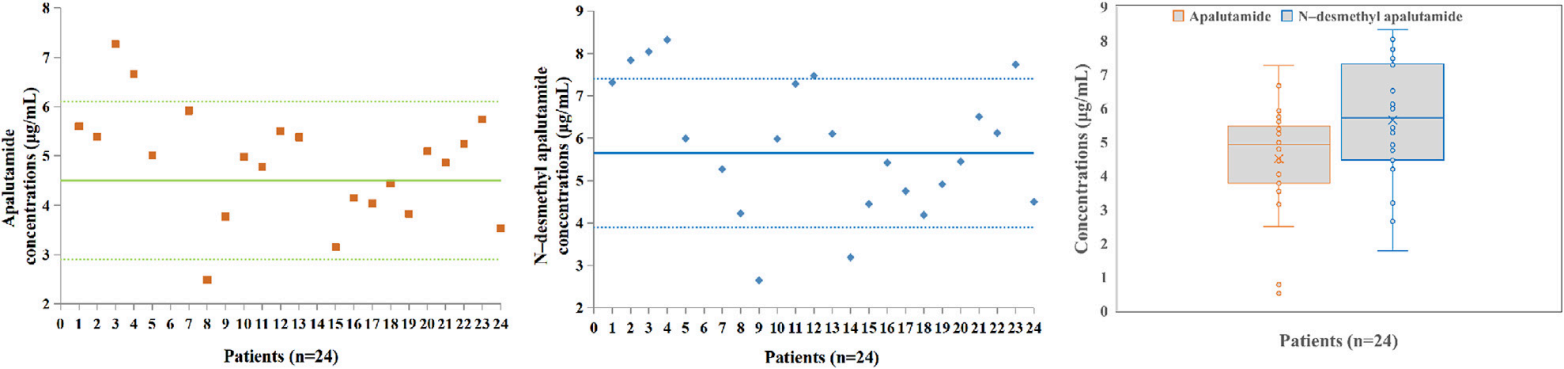
Apalutamide and N-desmethyl apalutamide plasma concentrations in patients (n = 24); box plot showing the spread of the apalutamide and N-desmethyl apalutamide concentrations measured in 24 patients’ plasma samples; data are presented as medians, with boxes representing the interquartile range (IQR 25th–75th percentile) and whiskers showing the minima and maxima.

The N-desmethyl apalutamide and apalutamide concentrations measured by applying the LC–MS/MS method in samples collected from patients receiving apalutamide for long-term treatment of NM-CRPC were all within the calibration range. A total of 24 blood samples from 24 patients with NM-CRPC were collected. The median age of the patients was 76 (range 53–94) years. Patients received regular oral doses of 180 mg once daily for at least 4 weeks, and trough concentrations were collected half an hour before dosing for blood concentration monitoring. The apalutamide (*p* = 0.065) and N-desmethyl apalutamide (*p* = 0.567) blood concentrations were normally distributed. The apalutamide blood concentration range was 0.517–7.27 μg/mL, and the median value was 4.92 μg/mL. The N-desmethyl apalutamide blood concentration range was 1.78–8.32 μg/mL, and the median value was 5.71 μg/mL. The ratio of N-desmethyl apalutamide to apalutamide concentration was 1.25. The results are shown in Figure 3.

**FIGURE 4:**
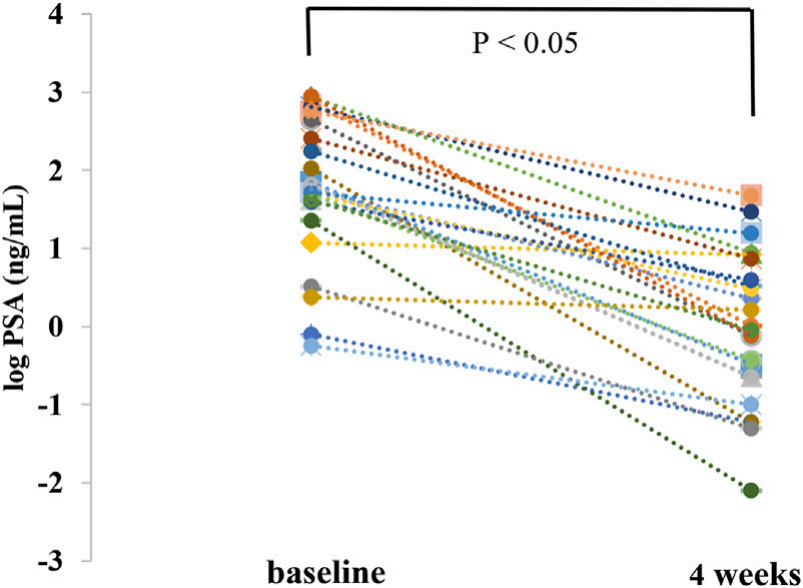
Comparison of prostate-specific antigen change from baseline at week 4 (PSA was corrected to log and then tested for normality).

The percentage change from baseline in the serum PSA level during treatment was significant. The median serum PSA level decreased from 61.03 (range 0.57–885.93) ng/mL at baseline to 0.970 (range 0.01–47.9) ng/mL at week 4. The median maximum reduction of percentage change from baseline in the serum PSA level during treatment was −97.98 (range −29.54 to −99.96%) (Table 4). As shown in Figure 4, the average log PSA at baseline was 1.76 ± 0.92 ng/mL, which was considerably greater than 0.00 ± 0.96 ng/mL of log PSA at week 4 (*p* < 0.05). The efficacy results of this study demonstrated a durable decline in PSA with once-daily dose of 180 mg of apalutamide, and patients taking 180 mg of apalutamide had a lower rate of rash than those taking 240 mg of apalutamide (T'Jollyn et al., 2022), which suggested that the drug dosage of 180 mg was still effective and safe.

**TABLE 4:** Summary of clinical characteristics of the patients taking apalutamide 180 mg (n = 24).

Age	
Mean (SD)	74.5
Median	76.0
Min, max	53, 94
Metastasis status, n (%)	
Bone metastasis	1 (4.2)
No bone metastasis	23 (95.8)
Rash grade, n (%)	
2	3 (12.5)
3	1 (4.2)
No rash	20 (83.3)
Total Gleason score, n (%)	
7	2 (8.3)
8	9 (37.5)
9	7 (29.2)
10	6 (25.0)
PSA concentration (ng/mL)	
Baseline, median (range)	61.030 (0.57–885.93)
Four weeks median (range)	0.970 (0.01–47.92)
Maximum reduction from baseline, median (range)	−59.755 (−885.15 to −0.47)
Maximum reduction of percent change from baseline, median (range)	−97.98 (−99.96 to −29.54)

PSA, prostate-specific antigen.

Apalutamide is rapidly absorbed after oral administration, with measurable plasma concentrations within 30 min. Peak plasma concentrations are achieved 2–3 h after administration, with a half-life of up to 7 days after a single oral dose. In addition, linear pharmacokinetics of apalutamide is across the 30–480 mg dose range, with plasma concentrations being dose proportional (Rathkopf et al., 2013). However, in our study, we have observed substantial individual variability in blood concentrations after a fixed dosage of 180 mg. The apalutamide and N-desmethyl apalutamide blood concentration ranges were 0.517–7.27 μg/mL and 1.78–8.32 μg/mL, respectively. This individual variation may be attributed to different age, physical conditions, or genetic polymorphism in drug metabolising enzyme. The greater the exposure, the more incidence of rashes, pruritus (May and Glode, 2019; Small et al., 2019; Smith et al., 2018), and seizures (Chi et al., 2019; Chi et al., 2021). Severe adverse drug effects caused by apalutamide may lead to drug discontinuation. Therefore, monitoring the blood concentration of apalutamide may promote efficacy and safety in its use in clinics.

Apalutamide is metabolised by CYP3A4 and CYP2C8 to its active metabolite, N-desmethyl apalutamide, which accounts for one-third of apalutamide’s pharmacological activity (T'Jollyn et al., 2022). Investigating active metabolites can elucidate the relationship between drug exposure to drug efficacy and adverse events. In our study, the blood concentrations of apalutamide and its active metabolite N-desmethyl apalutamide were simultaneously determined. The ratio of the steady-state trough concentration of N-desmethyl apalutamide to apalutamide was 1.25. Similar to our results, a previous study (Belderbos et al., 2018) reported that the ratio of N-desmethyl apalutamide to apalutamide steady-state peak concentration was 1.05, and the AUC24 h ratio was 1.33^13^.

Due to the effects of CYP3A4 and CYP2C8 on apalutamide metabolism (Duran et al., 2020), co-administration of apalutamide with strong inhibitors of these enzymes may necessitate dosage adjustments based on concentration results. Moreover, apalutamide is a strong inducer of CYP3A4 and CYP2C19 and a weak inducer of CYP2C9, P-gp, BCRP, or OATP1B1 in humans (Companies, 2019). Combining apalutamide with drugs that have sensitive substrates, such as CYP3A4, CYP2C19, CYP2C9, UGT, P-gp, BCRP, or OATP1B1, may result in decreased exposure. Therefore, drug substitution and loss of efficacy should be evaluated and dosages should be adjusted as needed during clinical use (Al-Salama, 2019; Posadas et al., 2020).

## 4 Conclusion

The primary objective of this study was to develop and validate a simple, specific, and effective method for the simultaneous determination of apalutamide and its active metabolite N-desmethyl apalutamide in human plasma using LC–MS/MS in the positive ion mode with MRM. This method showed acceptable precision and accuracy for quantifying apalutamide and N-desmethyl apalutamide in human plasma samples. It also complies with the guidelines for the validation of quantitative analysis methods for biological samples and is suitable for determining apalutamide concentration in human plasma. In this study, we provide valuable data on the steady-state trough concentration of long-term regular administration of apalutamide in actual clinical scenarios while capturing significant individual variability. Our results support further investigation into the relationship among apalutamide concentration, therapeutic efficacy, adverse drug reactions (ADRs), and patient characteristics. In addition, our results support for the rational use of clinical medication and defining PK targets for effective and safe treatment with apalutamide.

## Data Availability

The raw data supporting the conclusions of this article will be made available by the authors, without undue reservation.
